# Strong texture tuning along different crystalline directions in glass-supported CeO_2_ thin films by ultrasonic spray pyrolysis

**DOI:** 10.1038/s41598-021-81353-x

**Published:** 2021-01-21

**Authors:** Inti Zumeta-Dubé, José Manuel García Rangel, Jorge Roque, Issis Claudette Romero-Ibarra, Mario Fidel García Sánchez

**Affiliations:** 1 Instituto Politécnico Nacional, Centro de Investigación en Ciencia Aplicada y Tecnología Avanzada, Unidad Legaria, CDMX, Legaria 694, Col. Irrigación, 11500 Miguel Hidalgo, Mexico; 2 Unidad Profesional Interdisciplinaria en Ingeniería y Tecnologías Avanzadas, Instituto Politécnico Nacional, Av. I.P.N. 2580, Gustavo A. Madero, 07340 Mexico, Mexico; 3 CINVESTAV, Instituto Politécnico Nacional, Av. I.P.N. 2508, Gustavo A. Madero, 07340 Mexico, Mexico

**Keywords:** Materials science, Materials for energy and catalysis, Nanoscale materials, Structural materials

## Abstract

The strong facet-dependent performance of glass-supported CeO_2_ thin films in different applications (catalysis, smart windows, etc.) has been the target of diverse fundamental and technological approaches. However, the design of accurate, cost-effective and scalable methods with the potential for large-area coverage that produce highly textured glass-supported CeO_2_ thin films remains a technological challenge. In the present work, it is demonstrated that under proper tuning conditions, the ultrasonic spray pyrolysis technique enables one to obtain glass-supported polycrystalline CeO_2_ films with noticeable texture along both the (100) and (111) directions, as well as with randomly oriented crystallites (no texture). The influence of flow rates, solution molarity, and substrate temperature on the texture and morphological characteristics, as well as optical absorption and Raman response of the deposited films, is evaluated. The obtained results are discussed on the basis of the combined dependence of the CeO_2_-exposed surfaces on the thermodynamic stability of the corresponding facets and the reaction kinetics, which modulate the crystallite growth direction.

## Introduction

Cerium dioxide (CeO_2_), also known as ceria, is considered one of the most important rare earth oxides due to its multiple applications in several key areas of thin film technology^1,2^. In particular, nanostructured ceria thin films are very attractive due to their promising performance as catalysts for the treatment of automobile exhaust gases, where the catalytic activity is very sensitive to the exposed crystalline facets^3^. For instance, the (111) direction (an oxygen-rich surface) of the fluorite CeO_2_ structure is the most energetically stable, and, thus, the most frequently exposed facet in thin films, having an intrinsically low catalytic activity. However, the (100) direction (a cerium-rich surface) comprises a dipolar plane that is less stable, and, consequently, harder to grow in thermodynamic terms but more catalytically active than the (111) counterpart^3,4^. In contrast, under Pd surface modification of CeO_2,_ the (111) facet becomes more catalytically active than the (100) facet for room-temperature CO oxidation^5^. Regarding prospective applications in self-cleaning windows, the (111) surface is more hydrophobic than the (100) surface, while the (110) surface is the most hydrophilic^6^. This theoretical study also revealed that the relatively unstable oxygen-terminated (100) surface is stabilized by full coverage of molecular adsorbed water. Therefore, the surface properties of the (100) facet are critically dependent on the deposition method^6^. Therefore, strict control of the CeO_2_ growth direction (texture) in thin films is relevant for technological applications.

Several physical and chemical processes have been employed to prepare CeO_2_ thin films, including flash evaporation^7^, electron-beam evaporation^8^, sputtering^9^, laser ablation^10^, spin coating^11^ and spray pyrolysis^12–16^. Among these techniques, spray pyrolysis is very attractive for its low cost and effectiveness in the deposition of high-quality thin oxide films^17,18^. This technique also shows great versatility in both small- and large-area coverage, which makes it suitable for large-scale production methods^17,18^. Material selection (solvents, precursors, additives, substrates) and parameter optimization (molarity of solution, distance nozzle-substrate, flow rate, and spray generation) have a great influence on the final properties of films. Thus, the precise control of these parameters, as well as knowledge of their impact on the physicochemical phenomena involved in the corresponding chemical reactions, are crucial to control the morphology and material growth. When using pneumatic-based sprays to deposit CeO_2_ thin films on glass substrates, low crystalline films are typically obtained, and post-thermal annealing processes are frequently required^12^. Additionally, there is a great challenge in the accurate control of deposition parameters to obtain a strongly textured CeO_2_ film, either along the (111) or (100) directions, on such an amorphous substrate^12,19–21^. On the other hand, by using ultrasonic-based sprays, a small and homogeneous droplet size is obtained compared with pneumatic-based sprays, with a potential impact on film texture control and other physical properties^15–17^. Despite the potential of the ultrasonic spray pyrolysis (USP) technique, CeO_2_ thin film deposition on a glass substrate has rarely been explored^13^.

R. Suresh et al. used the USP technique to obtain CeO_2_ thin films, and the effects of water, ethanol and methanol solvents and the substrate temperature on the morphology of the deposit were studied^13^. Fairly inhomogeneous morphology and/or poor substrate coverage were observed in all cases. It was concluded that water and ethanol favour preferential orientation along the (200) reflection ((100) direction), while methanol favours the preferential orientation along the (111) direction. Temperatures near the extremes of the range between 300 °C and 500 °C produced amorphous films, while at intermediate temperatures, the X-ray diffraction patterns showed low crystalline features. Strikingly, discussion for the physicochemical origin of this behaviour was omitted, which could eventually lead to convenient modifications of the deposition route. A deep understanding of the growth mechanism is of relevant importance for film deposition experimental design. The impact of other important parameters, such as carrying flux and precursor concentration, was not included in the study by Suresh et al.^13^. As ultrasonic spray pyrolysis is highly dependent on many deposition parameters, more studies are required to fill some of the gaps that remain in previous work. On the other hand, García Sánchez et al. evaluated the influence of flow rates and substrate temperature on the structural, chemical and optical properties of CeO_2_ thin films prepared by USP using a system with two flow rates^16^. They found that flow rate variations modified the substrate temperature, crystalline orientation and Ce^3+^/Ce^4+^ ratio in the material. A mix of Ce^3+^ and Ce^4+^ was reported, even in samples with thermal annealing. A photoluminescence signal in the visible region of spectra was observed, which is associated with the high concentration of Ce^3+^ (~ 40% in as-grown samples and 25% in treated films), probably in the grain surface.

In the present work, the influence of flow rate, solution molarity and substrate temperature on texture and morphological characteristics, as well as on optical absorption and Raman response of nanostructured CeO_2_ thin films deposited by USD, are evaluated. The results demonstrate that tuning the proper deposition conditions allows us to obtain highly crystalline CeO_2_ films with strong texture along the (100) and (111) directions, as well as randomly oriented crystallites (no texture), on an amorphous substrate such as glass. Homogeneous substrate coverage can also be attained. The obtained results are discussed based on the reaction kinetics of crystallite growth. These findings are relevant for catalytic applications.

### Results and discussion

**Figure 1:**
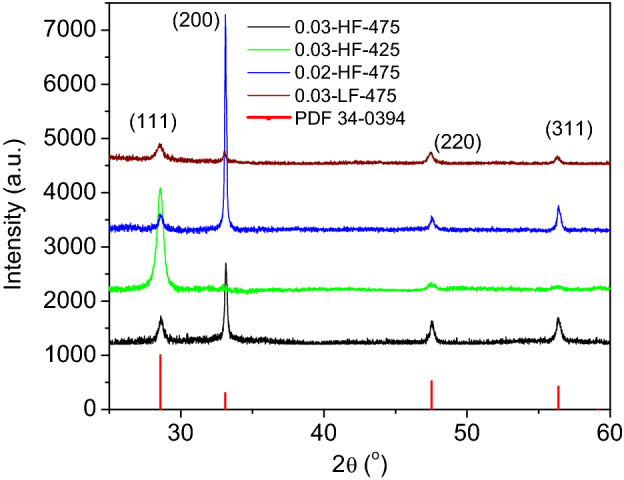
XRD patterns for the 0.03-HF-475, 0.03-HF-425, 0.02-HF-475, and 0.03-LF-475 samples; the PDF 34-0394 reference is included.

The XRD patterns for the 0.03-HF-475, 0.03-HF-425, 0.02-HF-475, and 0.03-LF-475 samples are presented in Fig. 1. Although growth took place on an amorphous substrate, the deposited films are polycrystalline, which is signed by the multipeak patterns. In all cases, the positions of the observed peaks match well with those of the PDF 34–0394 reference, corresponding to the fluorite structure (space group Fm3m) of CeO_2_ with a lattice constant of 5.41 Å. However, except for the 0.03-LF-475 sample, it is noteworthy that the relative intensities of the XRD patterns fairly deviate from those of the PDF 34-0394 reference, revealing well texturized CeO_2_ films. Regarding the sensitivity of this technique, no secondary phase impurities were detected.

The average crystallite sizes (D) were estimated from XRD patterns for the most intense peaks using the Scherrer formula^22^:

1 $$D=\frac{0.9\lambda }{\beta cos{\theta }_{B}}$$

Here, *λ* is the wavelength of the incident beam, *β* is the intrinsic width at half maximum of the *hkl* line and Θ_*B*_ is the Bragg diffraction angle. The preferential orientation can be evaluated by calculation of the texture coefficient (T_C_) using the formula^22^:

2 $$T_{{C_{{\left( {hkl} \right)}} }} = ~\frac{{{\raise0.7ex\hbox{${I\left( {hkl} \right)}$} \!\mathord{\left/ {\vphantom {{I\left( {hkl} \right)} {I_{0} \left( {hkl} \right)}}}\right.\kern-\nulldelimiterspace} \!\lower0.7ex\hbox{${I_{0} \left( {hkl} \right)}$}}}}{{\frac{1}{N}\mathop \sum \nolimits_{N} \left[ {{\raise0.7ex\hbox{${I\left( {hkl} \right)}$} \!\mathord{\left/ {\vphantom {{I\left( {hkl} \right)} {I_{0} \left( {hkl} \right)}}}\right.\kern-\nulldelimiterspace} \!\lower0.7ex\hbox{${I_{0} \left( {hkl} \right)}$}}} \right]}}$$

where $I\left(hkl\right)$ is the measured relative intensity of a plane $\left(hkl\right)$, ${I}_{0}\left(hkl\right)$ is the standard intensity taken from the PDF data and *N* is the number of diffraction peaks. T_C_ qualitatively evaluates the strength of the crystalline orientation along a certain (hkl) direction with respect to a randomly oriented PDF powder reference. As the T_C_ values are closer to unity, the crystallites of the film are more randomly oriented, while larger T_C_ values indicate stronger crystalline orientation along the corresponding (hkl) direction. The results for the crystallite size and T_C_ along the (111), (200), (220) and (311) directions are presented in Table 1.

**Table 1:** Crystallite sizes obtained using the Scherrer formula and the texture coefficients for the deposited CeO_2_ films.

Sample	Crystallite size (nm)				Texture coefficient			
	(111)	(200)	(220)	(311)	(111)	(200)	(220)	(311)
0.03-HF-475	17	41	20	21	0.4	2.2	0.5	0.9
0.03-HF-425	15	–	24	25	2.1	0.1	0.3	0.6
0.02-HF-475	20	72	40	44	0.2	3.1	0.2	0.5
0.03-LF-475	16	19	19	18	1.1	1.0	1.1	0.9

The obtained crystallite size along the (111) direction was observed to be the smallest in every sample, independent of the strength of the texture coefficient along this direction. For all samples, the Williamson-Hall approximation^23,24^ did not fit the relevant data obtained from the XRD experiment well. This fact means that crystallite size and/or lattice strain are fairly dependent of the crystalline direction in the films. This observation is supported by DFT calculations, as well as previous experimental works^25,26^. These works concluded that oxygen vacancies (which lead to lattice strain) become energetically favoured by Ce atom displacement along the (111) direction, resulting in a distorted Ce^3+/4+^-O tetrahedron. Therefore, the strain contribution to the peak broadening in the XRD pattern can significantly differ from one direction to another, being particularly strong along the (111) direction^25,26^. Thus, the crystallite size determination via the Scherrer formula should be read as a minimum and not necessarily as a value well adhered to the real size. When the crystallite sizes are compared with films grown on crystalline (silicon) substrates^16^, similar grain size values are obtained for the (111) direction, but higher values are obtained for the other directions. This could be related to the ceria tendency to grow in a columnar structure.

Table 1 shows that the 0.03-HF-475 sample is strongly texturized along the (200) direction, with a T_C_ of 2.2. It is well known that preferential growth of thin films derived from spray-based techniques is modulated by the substrate nature, reaction atmosphere (these two factors are common for the samples in this work), deposition temperature, precursor nature and concentration and carrying fluxes. These parameters could be interrelated in some way and affect the reaction kinetics. The reaction kinetics strongly influence (via the migration energy of the deposited atoms) the chance of ad-atoms accommodating (or not) minimal energy surfaces^24^. According to the surface energy of the different planes of the fluorite structure of CeO_2_, the surface stability of the crystal follows the order of (111) > (200) > (220) > (311)^24^. Therefore, the fact that the 0.03-HF-475 film is strongly texturized along the (200) direction means that the reaction kinetics under these conditions are fast enough (rapid crystallization) to inhibit ad-atom accommodation in the lower energy surface (111). Taking this sample (0.03-HF-475) as a reference, if a lower substrate temperature is used during the deposition process, as is the case for the 0.03-HF-425 sample, the texture coefficients become strong along the (111) direction (T_c_ = 2.1) and very weak along the remaining planes (T_C_ < 0.6). This result is indicative of a fairly slower reaction rate in these lower temperature conditions, which allows ad-atom migration to the favoured lowest surface energy facet (111) during crystal formation. In this case, the preferential growth along (111) is probably related to ordered staking of crystallites in this direction rather than large crystallite size (see Table 1). When a lower precursor concentration is used, such as that in the 0.02-HF-475 sample, the texture coefficient along the (200) direction becomes even stronger (but weaker along the remaining directions) than that of the 0.03-HF-475 counterpart (2.2 vs 3.1). This behaviour means that the reaction kinetics are even faster in this case, which can be understood since the energy available for the reaction to occur is the same for both the 0.02-HF-475 and 0.03-HF-475 samples, but the amount of organic matter to burn (giving rise to CeO_2_ formation) is smaller for the former. For the lower flux, such as that in the 0.03-LF-475 sample, a less texturized film is obtained, i.e., texture coefficients are close to the unit. This fact points to an intermediate kinetic reaction between 0.03-HF-475 and 0.03-HF-425. This means that lowering the flux decreases the reaction kinetics but to a lesser extent than lowering the substrate temperature (at least in the test conditions). In conclusion, in the explored parameter ranges, higher temperatures and fluxes and lower precursor concentration favour faster reaction kinetics, which trigger texture along the (200) direction, while lower temperature and flux, as well as higher precursor concentration, favour slower reaction kinetics, promoting texture along the (111) direction. This result overcomes preceding studies regarding texture control of CeO_2_ thin films on glass substrates by USD^13^, but it is consistent with ceria grown on silicon substrates^16^. This is an interesting result that suggests that texture is more influenced by the control of grown conditions than by the nature of the substrate.

**Figure 2:**
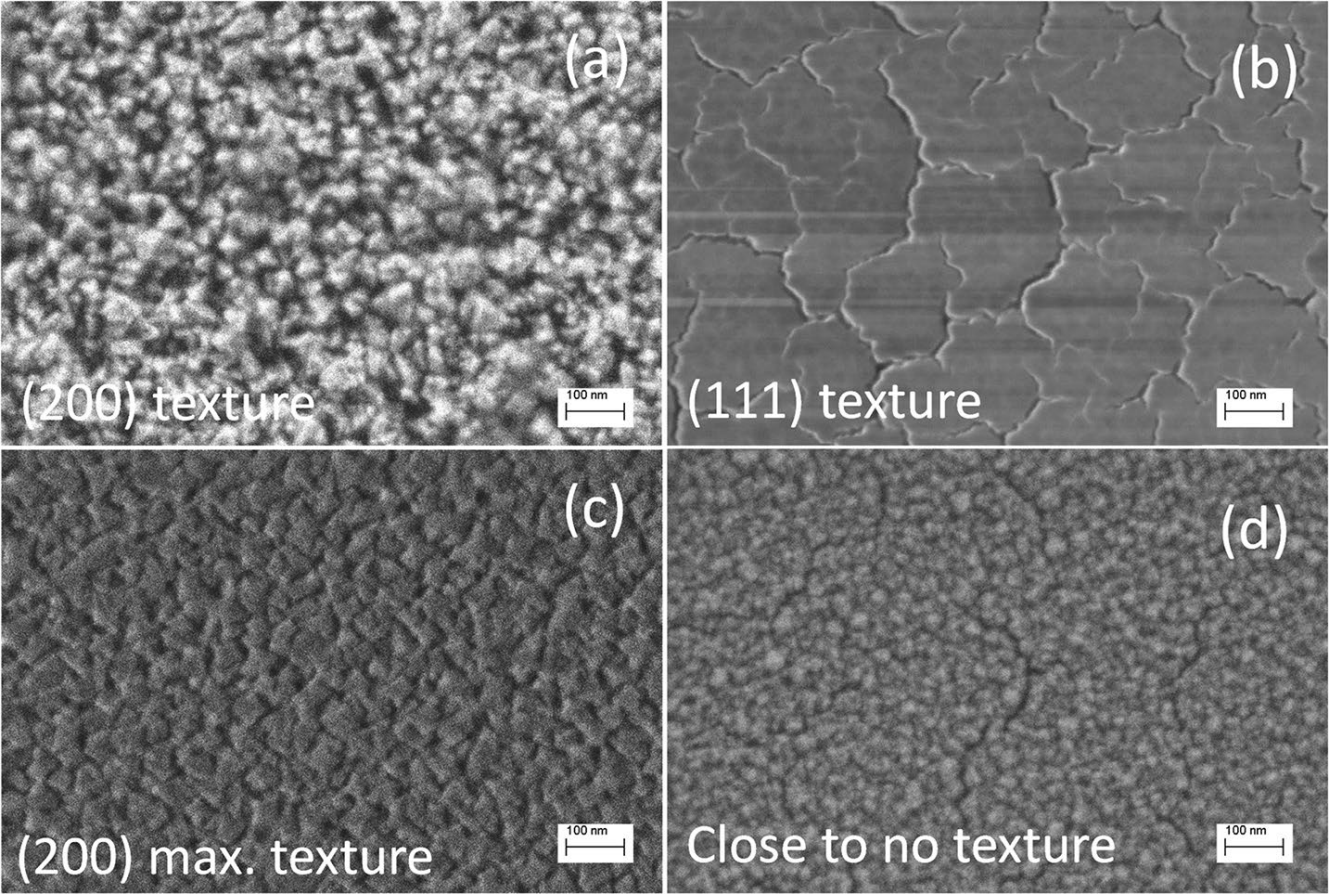
SEM micrographs of the (**a**) 0.03-HF-475, (**b**) 0.03-HF-425, (**c**) 0.02-HF-475, and (**d**) 0.03-LF-475 samples.

SEM micrographs of the 0.03-HF-475, 0.03-HF-425, 0.02-HF-475, and 0.03-LF-475 samples are shown in Fig. 2. The surface morphology of the layers observed by SEM is a consequence of the degree of crystallinity and orientation (texture) resulting from the growth process. Therefore, the SEM technique allows visualization of the impact of texture on the morphological termination of the surface of the films. Correlating TCs with morphological surface characterization by SEM for films is a very useful and common characterization strategy^27^. Thus, the discussion delivered above for the origin of film texture can be extrapolated here to the corresponding morphology observed by SEM.

SEM micrographs of the 0.03-HF-475 sample show homogeneous coverage of the substrate (Fig. 2a). The sample has a rough surface morphology composed of irregular polygonal grains with sizes ranging from ~ 20 to 50 nm. We want to note that this film presented a (200) texture (Table 1). In contrast, the 0.03-HF-425 sample (with a strong texture along (111)) has a fairly flat surface morphology with a crack network delimiting islands of ~ 100–300 nm in size (Fig. 2b). It is known that vacancy accumulation favours film cracking while minimizing the lattice strain and energy^28^; thus, this morphology is in accordance with the preferred strain accumulation (probably originating from oxygen vacancy formation) along the (111) direction, as hypothesized above^26^. Considering the intrinsic anisotropic nature of the CeO_2_ structure and some related properties^25^, an anisotropic thermal expansion coefficient showing a larger mismatch along (111) with respect to that of the substrate cannot be discarded. There are no reports in the literature in this regard. The morphology of the 0.02-HF-475 sample (Fig. 2c) somehow resembles that of the 0.03-HF-475 sample, but in this case, the surface is more homogeneously covered, and the polygonal grains are better defined, packaged and show sharper edges. In correspondence with the analysis above, this behaviour is associated with the stronger texture along the same (200) direction. For this sample, grain sizes are also in the ~ 20–50 nm range. The morphology of the 0.03-LF-475 sample (Fig. 2d) is intermediate between that of the 0.03-HF-475 and 0.03-HF-425 samples. This result is also in accordance with the observations from the XRD analysis.

**Figure 3:**
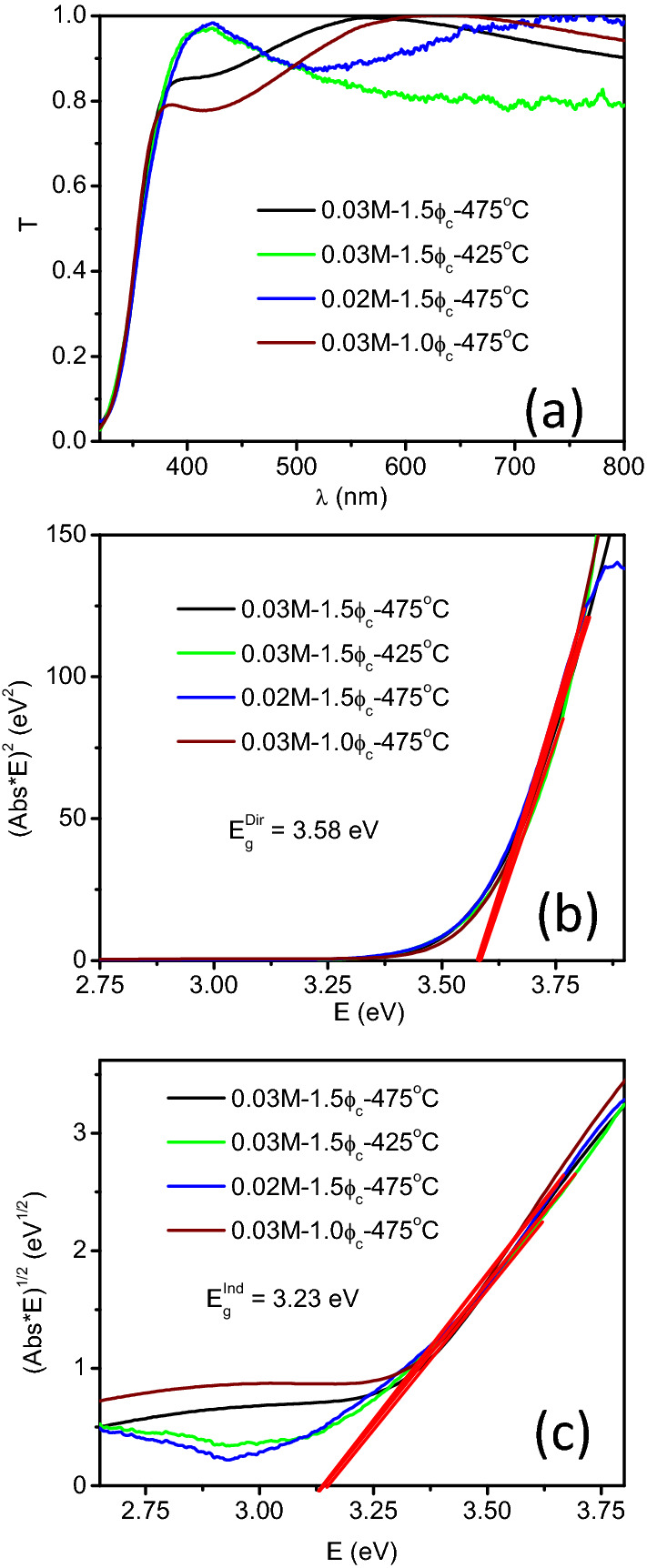
(**a**) Transmittance spectra and the corresponding Tauc plots for the direct (**b**) and indirect (**c**) gaps for the 0.03-HF-475, 0.03-HF-425, 0.02-HF-475, and 0.03-LF-475 samples.

The transmittance spectra for the 0.03-HF-475, 0.03-HF-425, 0.02-HF-475, and 0.03-LF-475 samples are presented in Fig. 3a. The spectral onset (below ~ 390 nm) for all the studied samples is very similar. The spectral oscillations in the ~ 390–800 nm range obey the interference effect, as typically observed in transparent thin films. Different thicknesses lead to different oscillation amplitudes.

The corresponding Tauc plots for the data in Fig. 3a for direct and indirect band gap calculations are shown in Fig. 3b and c, respectively. All the samples have the same direct band-gap energy (E_g_^Dir^) value of 3.58 eV and indirect band-gap (E_g_^Ind^) of 3.23 eV. There are two gap values reported for bulk CeO_2_: an indirect gap in the 2.99–3.3 eV range and a direct gap at 3.57 eV^29,30^. Thus, the obtained E_g_ values are fairly coincident with those reported for bulk CeO_2_ and are also in accordance with the transparent appearance of the layers. It has been reported that the absorption edge of ceria films is not significantly altered up to doping levels of 15% substitution of Ce^+4^ by trivalent cations^31^. The coincidence between the obtained E_g_ indicates that all the samples studied in this work present similar electronic structures (at least regarding the top of the valence band and the bottom of the conduction band). This fact is the cause of the observed similar spectral onset in Fig. 3a. These E_g_ values define the electronic transitions from the O 2p-based top of the valence band to the partially occupied bottom of the conduction band, which is dominated by the Ce 4*f* orbital contribution^32^.

**Figure 4:**
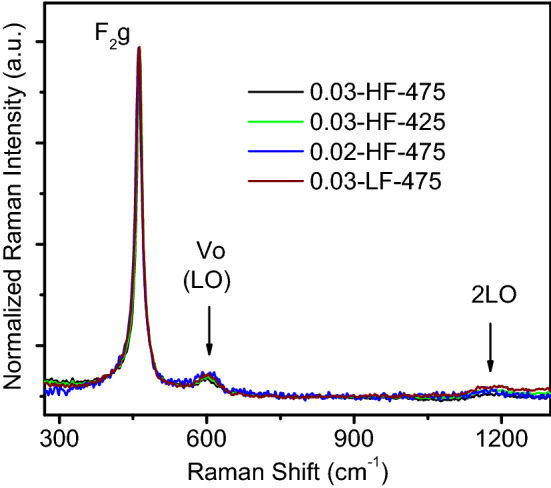
Raman spectra for the 0.03-HF-475, 0.03-HF-425, 0.02-HF-475, and 0.03-LF-475 samples.

As Raman scattering spectroscopy is a powerful technique in the structural characterization of materials, it was used for further analysis. The Raman spectra for the 0.03-HF-475, 0.03-HF-425, 0.02-HF-475, and 0.03-LF-475 samples are shown in Fig. 4. The four spectra profiles are fairly coincident (within 5%) and show characteristic features corresponding to the fluorite structure (Fm3 m) of CeO_2_. As in XRD analysis, only CeO_2_ was detected in all cases.

The most intense band is located at approximately 462 cm^−1^ and is attributed to symmetric stretching of the Ce^4+^-O bonds (F_2g_ symmetry) of the Ce-O8 octahedron^5^. This band is redshifted by 4 cm^−1^ with respect to that of the stoichiometric single crystal^5^. This observed redshift is ascribed to a substoichiometric composition of CeO_2−δ,_ which can be roughly estimated as δ = 0.023 by following the methodology developed in^33^. By means of a semiempirical model^32^, the F_2g_ band also allows an estimation of the average crystallite size, correlation length (homogeneity size), and order of magnitude of oxygen vacancy density of 30–60 nm (comparable to that obtained from XRD), 18–10 nm and 10^17^–10^18^ cm^−3^, respectively. The weak band at approximately 590 cm^−1^ corresponds to a longitudinal optical (LO) Raman-forbidden mode (F_1u_ symmetry). This band becomes Raman-active due to oxygen vacancy defects, which leads to a lack of symmetry, and, consequently, relaxation of the selection rules^34^. The other weak band at approximately 1180 cm^−1^ is the corresponding 2LO overtone with A_1g_ symmetry^34^.

The Raman spectrum of a given material is ultimately determined by its structure and symmetry and is sensitive to structural defects and crystallite size (depending on the excitation conditions and sample nature)^32–34^. Thus, the observed similarity of the four Raman spectral shapes obtained in this work points to similar bulk structure, symmetry and defect nature and densities in the studied films, at least up to the sensitivity of the excitation conditions used in this work (laser wavelength and power of 455 nm and 7 mW, respectively, for the best signal-to-noise ratio). It needs to be mentioned that to more accurately sense the presence of vacancies, crystallite frontiers, or other structural discontinuities of the lattice periodicity, it is preferable to measure the Raman active modes while electron transfer from the O 2*p* to Ce 4*f* bands takes place, i.e., in the resonance condition^34^. The spectra were collected with a blue laser (455 nm), and a UV laser was required (photon energy above the corresponding computed E_g_). This condition allows the preferential amplification of oxygen vacancy-related modes and eventually establishes a major spectral differentiation between the studied films.

## Conclusions

The physicochemical-based selection of critical deposition parameters in the ultrasonic spray pyrolysis technique allows precise control of the morphology and texture of polycrystalline CeO_2_ thin films on glass substrates. These results improve those of a previous study^13^. Considering the following deposition parameter ranges: molarity of 0.02–0.03 M, carrier flow of 1.0–1.5 L min^−1^, directing flow of 1.5–2.0 L min^−1^ and substrate temperature of 425–475 °C, increasing the temperature and flux and decreasing the precursor concentration favours fast reaction kinetics, which promote texture along the (200) direction. On the other hand, decreasing the temperature and flux as well as increasing the precursor concentration favours slow reaction kinetics, resulting in texture along the (111) direction. This is relevant not only for being the first report of this specific concern but also for facet-dependent applications of supported CeO_2_. The resulting films are strongly anisotropic regarding certain structural and morphological features but fairly homogeneous in their absorption and vibrational response, given the sensitivity of the used techniques. These results are very relevant considering the increased interest in ceria for catalytic and smart window applications.

## Materials and methods

Films were grown with the experimental setup previously reported^17^. It is important to note the use of two different flow rates in this USP system. A carrier gas transports the mist from the nebulizer to the nozzle. The maximum flow rate allowed for this gas is related to the mist generation rate to maintain the continuity of the deposition process. However, it is very important to maintain a quasi-laminar flow, as turbulence can produce a coarsening of drops, which decreases the homogeneity of the deposition process. On the other hand, the director gas enables an increase in the kinetic energy of droplets arriving on the heated substrate but in the same way reduces the time required for droplets to approach the substrate^16^. Films were deposited onto glass slices, which were ultrasonically cleaned in different steps with trichloroethylene (C_2_HCl_3_), acetone (C_3_H_6_O) and isopropanol (C_3_H_8_O). The spray solution was cerium(IV) acetylacetonate [Ce(acac)_4_ = Ce(C_5_H_7_O_2_)_4_] from Sigma-Aldrich Chemicals dissolved in anhydrous methanol. One mL of acetic acid (CH_3_COOH) in 0.5 L of anhydrous methanol was added^15^. Table 2 summarizes the four different experimental conditions used in this work. These conditions were selected to evaluate the influence of three different growth parameters (solution molarity, flow rates and substrate temperature). The first sample (0.03-HF-475) was prepared with a precursor concentration of 0.03 M, substrate temperature of 475 °C and flow rates of 1.5 L min^−1^ and 2.0 L min^−1^ for the carrier and directing gas, respectively. For the second sample (0.02-HF-475), the molarity was varied (0.02 M) with regard to the first sample. For the third sample (0.03-LF-475), the flow rates were changed: low flow (LF) rates of 1.0 L min^−1^ and 1.5 L min^−1^ were used instead of high flow (HF) rates of 1.5 L min^−1^ and 2.0 L min^−1^ for the carrier and directing gas, respectively, in the first sample. Finally, 425 °C was used as the heating plate temperature for the four samples (0.03-HF-425), instead of the temperature of 475 °C used in the first sample. The deposition time was fixed at 60 min, and air was used as the carrier and directing gas for all thin films.

**Table 2:** Experimental conditions for the deposition of CeO_2_ films on glass substrates.

Sample	Molarity (M)	Carrier gas flow rate (L min^−1^)	Director gas flow rate (L min^−1^)	Substrate temperature (^o^C)
0.03-HF-475	0.03	1.5	2.0	475
0.02-HF-475	0.02	1.5	2.0	475
0.03-LF-475	0.03	1.0	1.5	475
0.03-HF-425	0.03	1.5	2.0	425

### Characterization techniques

X-ray diffraction patterns (XRD) were obtained using a D2 PHASER diffractometer (from Bruker) with a Cu source (Kα1 radiation, λ = 1.5406 Å) operating at 10 mA and 30 kV in a Bragg–Brentano configuration. The transmittance spectra for the films were collected using a Cary-5000 spectrometer from Agilent. Raman scattering spectroscopy was performed using a DXR Micro-Raman spectrometer from Thermo Fisher Scientific with an excitation laser of 455 nm at 7 mW of power by focusing the laser radiation through a 50X microscope objective lens at 0.2 mW using 5 s as the integration time and 3 scans. Films were examined with a Zeiss field emission SEM (AURIGA model) operating at 1 kV.
